# Quantifying redox-induced Schottky barrier variations in memristive devices via *in operando* spectromicroscopy with graphene electrodes

**DOI:** 10.1038/ncomms12398

**Published:** 2016-08-19

**Authors:** Christoph Baeumer, Christoph Schmitz, Astrid Marchewka, David N. Mueller, Richard Valenta, Johanna Hackl, Nicolas Raab, Steven P. Rogers, M. Imtiaz Khan, Slavomir Nemsak, Moonsub Shim, Stephan Menzel, Claus Michael Schneider, Rainer Waser, Regina Dittmann

**Affiliations:** 1 Peter Gruenberg Institute, Forschungszentrum Juelich GmbH and JARA-FIT, 52425 Juelich, Germany; 2 Institute of Materials in Electrical Engineering and Information Technology II, RWTH Aachen University, 52056 Aachen, Germany; 3 Department of Materials Science and Engineering and Materials Research Laboratory, University of Illinois, Urbana, Illinois 61801, USA

## Abstract

The continuing revolutionary success of mobile computing and smart devices calls for the development of novel, cost- and energy-efficient memories. Resistive switching is attractive because of, inter alia, increased switching speed and device density. On electrical stimulus, complex nanoscale redox processes are suspected to induce a resistance change in memristive devices. Quantitative information about these processes, which has been experimentally inaccessible so far, is essential for further advances. Here we use *in operando* spectromicroscopy to verify that redox reactions drive the resistance change. A remarkable agreement between experimental quantification of the redox state and device simulation reveals that changes in donor concentration by a factor of 2–3 at electrode-oxide interfaces cause a modulation of the effective Schottky barrier and lead to >2 orders of magnitude change in device resistance. These findings allow realistic device simulations, opening a route to less empirical and more predictive design of future memory cells.

## Abstract (web summary)

Resistive switching in metal oxides is related to the migration of donor defects. Here Baeumer *et al.* use *in operando* X-ray spectromicroscopy to quantify the doping locally and show that small local variations in the donor concentration result in large variations in the device resistance.

## Graphical abstract

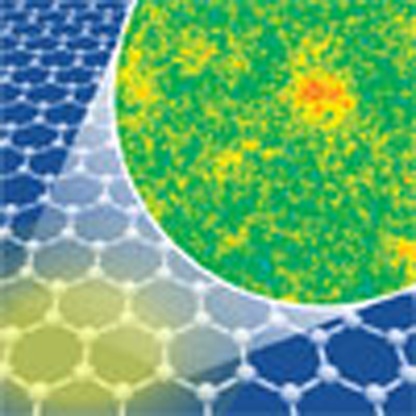

Since the rediscovery of resistive switching in transition metal oxides at the end of the 1990s (ref. 1), excellent device performance has already been established by many academic and industrial groups^2^,^3^. There exists a general consensus that resistive switching in transition metal oxides can be attributed to the motion of mobile donor-type defects, such as oxygen vacancies, and the corresponding valence change in the transition metal cation^4^. The quantitative details of the related nanoscale redox processes, however, are still elusive due to the lack of analytical methods which provide information about the electronic structure with sufficient spatial resolution and sensitivity to detect small variations. On the other hand, simulations have shown that even small modulations of the oxygen-vacancy concentration can induce significant changes of the charge–carrier transport and the resistivity of the memristive devices^5^,^6^,^7^,^8^,^9^. Therefore, to improve the predictive power of device simulations, which will ultimately improve the device design and performance, experimental advances in the characterization of nanoscale electronic structure changes during switching are critical prerequisites.

A variety of spectroscopic studies have identified the formation of an oxygen-vacancy-rich filament during the first biasing step, the so-called forming step^10^,^11^,^12^,^13^ and subsequent switching steps^14^,^15^. However, despite remarkable success thus far, these studies have been limited to non-functioning, post-mortem devices (for example, after top electrode removal^12^,^14^) due to the surface sensitivity of photoelectrons. This approach, of course, makes the examination of the same device in both resistance states inaccessible. At the same time, non-destructive, *in operando* spectroscopy techniques using hard X-rays^16^ or transmission X-ray microscopy^17^,^18^—while providing insightful information about the role of Joule heating and failure mechanisms—have failed to supply spatially resolved, quantitative spectroscopic differences between different resistance states. Spectroscopic characterization of the same switching filament in each resistance state, however, is mandatory for a quantitative description of the switching effects. This quantification, in turn, can finally yield a comprehensive understanding of memristive devices for future memory or logic applications. Therefore, a combination of the successful but previously destructive photoelectron emission microscopy (PEEM) studies, which allow for spectroscopic evaluation with high spatial resolution and interface- or surface sensitivity, with a non-destructive *in operando* approach is highly desirable.

The main challenge in the implementation of *in operando* PEEM characterization is to overcome the high surface sensitivity of photoemission, which limits the probing depth to few nanometers and practically prevents access to the active region covered by top electrodes. Graphene is not only highly conductive, but is also highly photoelectron-transparent and, when used as top electrode, may circumvent this limitation. Graphene electrodes have already been integrated successfully in memristive devices^19^,^20^, demonstrating improved cycling reproducibility and the potential for low-power operation and (photon)-transparent memories. They have even been found to enable ultra-high-density, cost-effective three-dimensional ReRAM arrays^21^.

Here we used uncovered single layer graphene as the top electrode for SrTiO_3_-based memristive devices, allowing simultaneous electrical biasing and imaging inside PEEM instruments. Due to well-known defect chemistry^22^ and diffusion properties^23^, SrTiO_3_ thin-film devices commonly serve as single-crystalline resistive-switching model system^4^. We find that graphene scarcely dampens the photoelectron intensity of buried layers, making it the ideal electrode material to study buried layers using surface sensitive spectromicroscopy. More importantly, we were able to achieve comprehensive understanding of the microscopic processes during the resistance change. During switching, the X-ray absorption signature of the switching filament changes reproducibly. These spectral changes correspond to spatially confined changes of the donor concentration by a factor of 2–3 at the graphene/SrTiO_3_ interface, causing changes of >2 orders of magnitude in the device resistance. Nanoionic device simulations show that this change in resistance is induced by the modulation of the effective Schottky barrier height and width. The unprecedented, quantitative agreement between experimental quantification of localized redox reactions and device simulation clarifies the microscopic changes on electrical stimulus and paves the way for implementation of memristive devices for future memory and logic applications.

## Results

### Graphene as photoelectron-transparent electrode

**Figure 1:**
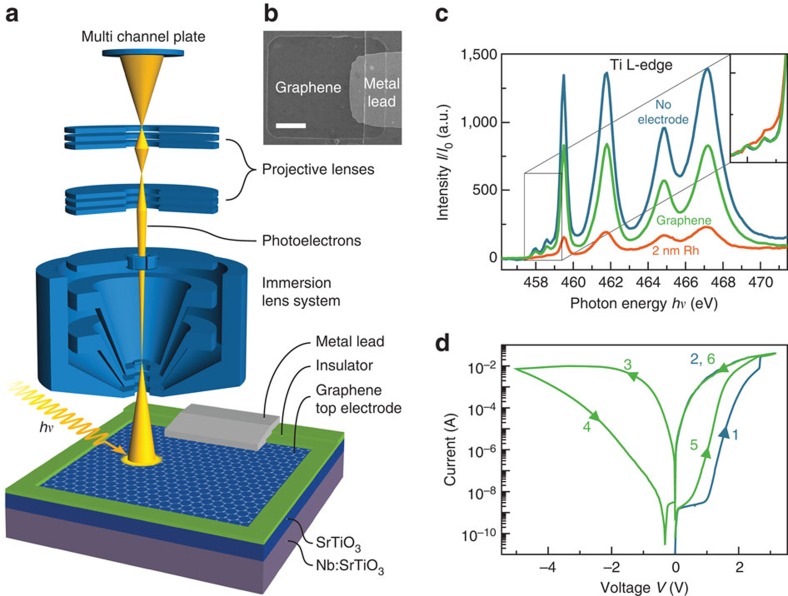
Graphene electrodes for *in operando* spectromicroscopy of memristive devices. (**a**) Device and measurement set-up schematic. An epitaxial SrTiO_3_ layer (blue) is sandwiched between a Nb:SrTiO_3_ bottom electrode (violet) and graphene top electrode (grey honeycomb lattice). The graphene electrode is contacted through a metal lead, which is electrically separated from the continuous bottom electrode, allowing for biasing inside PEEM instruments. At the same time, photoelectrons from the SrTiO_3_ layer can easily escape through the graphene electrode, allowing simultaneous imaging, as depicted with the PEEM lens system. (**b**) Scanning electron microscopy image of an exemplary device. Scale bar, 5 μm. (**c**) SrTiO_3_ Ti L-absorption edge detected from SrTiO_3_ with a 1 nm Al_2_O_3_ capping layer without electrode, from underneath a graphene electrode, and from underneath a 2 nm Rh electrode. Inset: zoom to the prepeak-area after normalization confirming better spectral resolution with graphene electrodes compared with Rh electrodes. Normalized spectra from underneath graphene and from the surface without electrode are practically identical. (**d**) Forming step (blue line) and following reset-set (green line) operation for a device with a graphene top electrode.

To achieve the desired *in operando* PEEM characterization of the resistive switching process, we employed SrTiO_3_-based memristive devices with graphene top electrodes (Fig. 1a,b). In a first step, to confirm that a sufficient photoelectron signal from the SrTiO_3_ thin film can be obtained through graphene layers, we compared the absolute electron yield at the Ti L-edge for a SrTiO_3_ thin film without electrode with a SrTiO_3_ thin film covered by a graphene electrode and a film covered by a 2 nm Rh electrode at comparable experimental conditions. Compared with the SrTiO_3_ without electrode, the photoelectron signal underneath the Rh electrode is diminished by a factor of 7 (Fig. 1c). Thus, the investigation of buried layers underneath ultra-thin Rh metal is in principle possible but very difficult due to low signals. Utilizing graphene electrodes, on the other hand, allows for a much stronger signal, which is only reduced by a factor of 1.6. In contrast to the spectrum from the film covered by Rh, even the small prepeaks on the low-energy side of the L_3_ edge are well-reproduced, confirming that spectromicroscopy using graphene electrodes is sensitive even for subtle spectroscopic changes of the buried layer (Fig. 1c, Inset). This electron-transparency is the reason why secondary electron microscopy and X-ray photoelectron spectroscopy through graphene membranes are possible^24^,^25^,^26^.

Going beyond pure membrane-applications, we also utilized the graphene layer as top electrode material, thus combining the beneficial effects of its single-atom thickness and excellent electrical conductivity. Electrical characterization of memristive devices with graphene electrodes confirms similar switching characteristics compared with devices with noble metal top electrodes (Fig. 1d). We observe the typical resistive switching where positive voltages applied to the top electrode set the device to the low-resistance state (LRS) and negative voltages reset the device to the high-resistance state (HRS). Based on these findings, we conclude that graphene electrodes with their unparalleled conductance-to-thickness ratio^21^ are the ideal electrode material to study buried layers using spectromicroscopy.

### Quantifying charge–carrier density modulations

**Figure 2:**
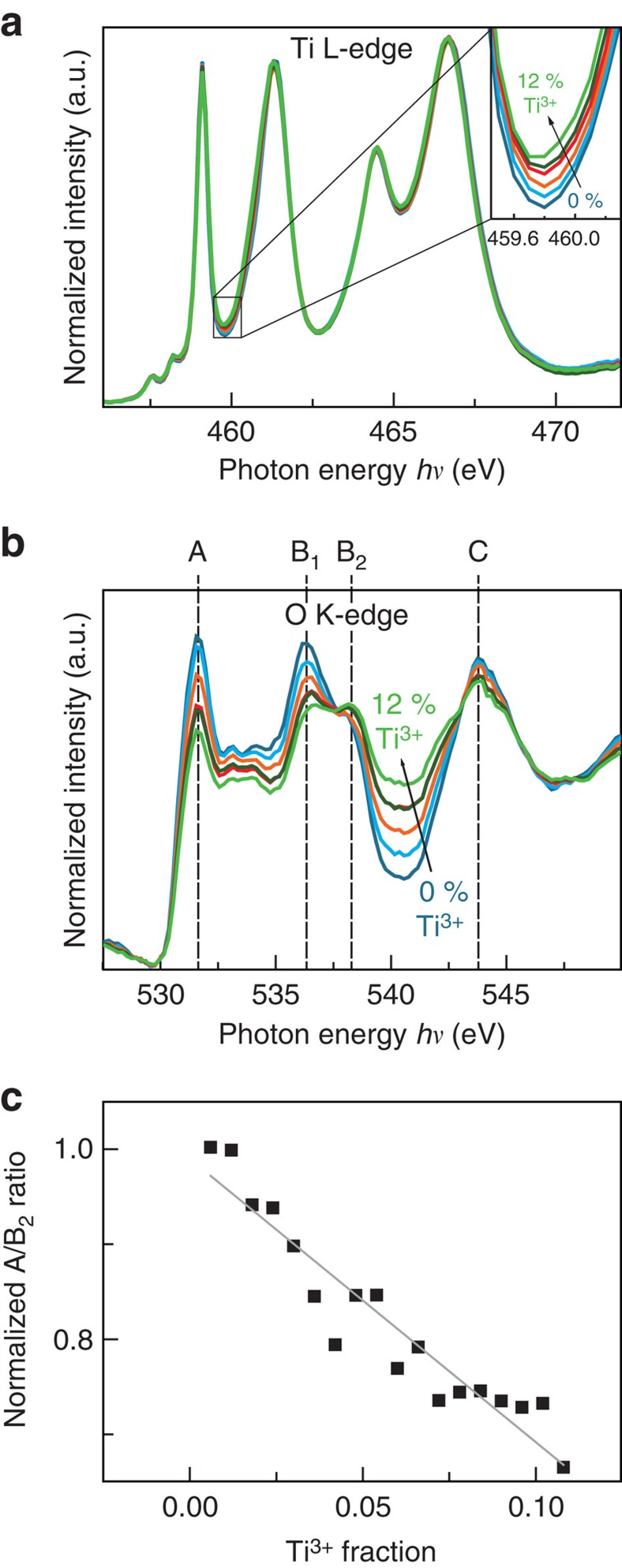
Calibrating X-ray absorption spectroscopy for the detection of subtle changes in oxygen-vacancy concentration. (**a**) Ti L-edge for increasing Ti^3+^ concentrations (0–11%). Inset: zoom to the photon energy of the Ti^3+^ L_3_ e_g_-edge. (**b**) O K-edge for the same Ti^3+^ concentrations. (**c**) Normalized A/B_2_ peak ratio for different Ti^3+^ concentrations. Grey line is a linear fit to the data and serves as a calibration curve.

Since even minor changes in the electronic structure are expected to have a large impact on the resistivity, we carefully evaluated the accessible X-ray absorption edges to resolve small changes during switching. It is well-known that oxygen vacancies in SrTiO_3_ lead to Ti^3+^ states (i.e., partial filling of the conduction band), which can be easily detected in the Ti L-edge if the Ti^3+^ concentration is ≥10–20% (refs 27, 28). At the same time, we expect changes in the O K-edge to occur^27^,^29^. To compare which absorption edge is more sensitive to small changes in the oxygen-vacancy concentration, we used XPEEM (PEEM operated in absorption mode with X-ray excitation) to extract spectra from an area of reduced SrTiO_3_ with different amounts of Ti^3+^ (Supplementary Fig. 1). As expected, there are significant changes in the Ti L-edge spectrum for high Ti^3+^ concentrations. The L_3_ e_g_ peak is broadened and shifted to lower photon energies for increasing amounts of Ti^3+^. In comparison, the spectral changes for small Ti^3+^ concentrations (<10%, Fig. 2a), are only very subtle in the Ti edge spectra, which we were only able to identify through numerical least-square fitting of each spectrum as a linear combination of reference spectra. If we consider the O K-edge spectra for the same regions of small Ti^3+^ concentrations (Fig. 2b), spectral changes are much more pronounced. The most obvious trend with increasing Ti^3+^ concentration is the decrease of the peak at a photon energy of 531.6 eV (referred to as peak A in the following) and an increase in the intensity of the valley between peaks B_2_ and C. Peak A corresponds to a transition from the O 1s level to a hybrid level between O 2p and Ti t_2g_ states. This decrease can, therefore, be understood as a decrease in the number of unoccupied hybridized O 2p-Ti t_2g_ states available for the transition from the O 1s level, that is, decreased X-ray absorption probability at this photon energy (compare ref. 30 and Supplementary Note 1). Therefore, a decreasing peak A intensity with increasing number of electrons in the conduction band can be expected^30^,^31^. In fact, we observe a nearly linear trend of the A/B_2_ peak intensity ratio for Ti^3+^ concentrations <10% (Fig. 2c), which yields a distinct calibration for the purpose of this work. We note that the same trend in the A/B_2_ ratio can be reproduced through annealing of SrTiO_3_ thin films in vacuum (Supplementary Fig. 2). As a result of defect equilibria, an increased concentration of donor-type oxygen vacancies is expected for increasing annealing temperature. Due to the pronounced changes appearing in the O K-edge in the small carrier concentration regime, the O K-edge is more sensitive to the anticipated changes of the electronic structure during resistive switching than the commonly used Ti L-edge. Consequently, we use the O K-edge for quantification of the electronic structure.

**Figure 3:**
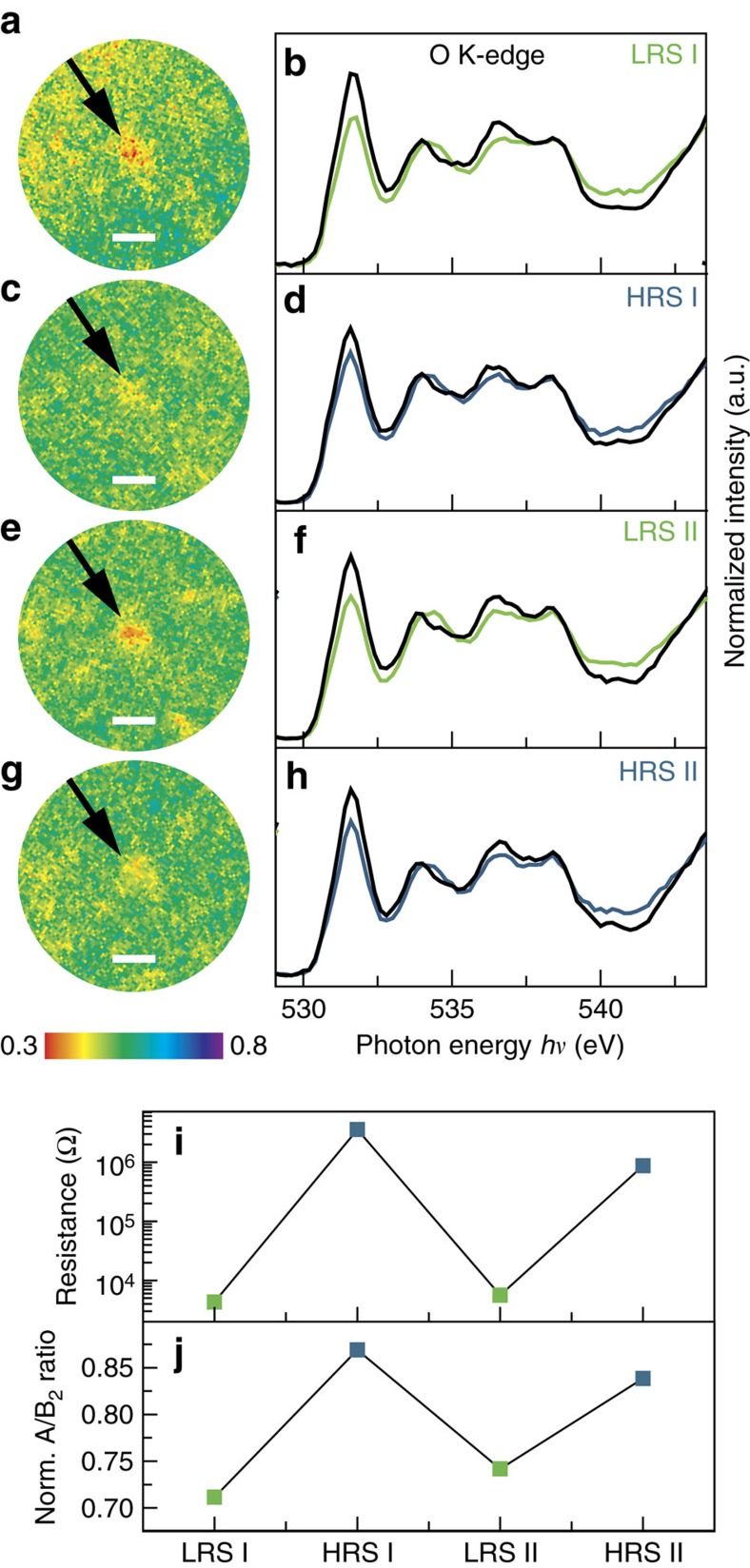
Spectromicroscopic quantification of resistive switching filaments. (**a**,**c**,**e**,**g**) PEEM images of the switching filament in the LRS, HRS, LRS II and HRS II, respectively (indicated by the black arrow) for a photon energy of 531.6 eV (peak A). Scale bars, 1 μm. (**b**,**d**,**f**,**h**) O K-edge for the switching filament in **a**,**c**,**e**,**g** (green and blue lines for the LRS and HRS, respectively) and the surrounding device area (black lines). (**i**) Device resistance and (**j**) normalized A/B_2_ ratio as a function of the device state.

Given these spectral signatures, we now turn to spectromicroscopic evaluation of functioning devices. For this purpose, we investigated the same device during a switching cycle LRS→HRS→LRS→HRS by acquiring O K-edge image stacks after each switching event. On close examination of the entire device area, we found a region-of-interest (ROI) exhibiting reduced intensity in the LRS at peak A (red spot in Fig. 3a). Extracting the entire O K-edge spectrum for this ROI and the surrounding area reveals the fingerprint of reduced SrTiO_3_ for the ROI (Fig. 3b). The same ROI shows much weaker contrast at peak A in the HRS (Fig. 3c), which leads to the suspicion that this region is a switching filament. For quantification, we extracted the O K-edge spectra for this filament in each resistance state (Fig. 3a–g), which show a reproducible change in the normalized intensity of peak A. This change in intensity, in turn, exhibits a direct correlation with the device resistance (Fig. 3i,j). To verify that this region is in fact the active switching filament, we also analysed all other regions-of-interest in the entire device area, which show contrasts in the O K-edge, but could not find significant changes on electrical stimulus. We, therefore, conclude that we found the fingerprint of a single active switching filament which shows a large number of carriers at the conduction-band edge in the LRS, and a lower concentration in the HRS. Comparison to the reference established in Fig. 2 yields approximate values of 9±2% Ti^3+^ in the LRS and 4±1% Ti^3+^ in the HRS. As XPEEM is a highly surface sensitive technique, the resulting charge–carrier densities of 1.5 × 10^21^ electrons per cm^3^ (LRS) and 6.7 × 10^20^ electrons per cm^3^ (HRS) can be considered as an average value of the top 2–3 nm of the filament. In the lateral direction, we estimate an upper limit of 500 nm for the diameter of this filament (this was found to be the necessary size of the ROI used to extract the spectra). Although experimental uncertainties limit the accuracy of assigning absolute values, these results provide valuable quantitative information on the charge–carrier density differences between different resistance states. They finally yield direct evidence that resistive switching in transition metal oxides is driven by a nanoscale redox reaction: an oxygen-vacancy-driven valence change in the Ti leads to a charge–carrier density modification by a factor of 2–3. Beyond a comprehensive understanding of the physicochemical switching mechanism, this information can complement existing device simulation models, enabling unprecedented predictive modelling approaches for future memory or logic devices.

### Spectroscopic quantities as input for nanoionic device simulations

**Figure 4:**
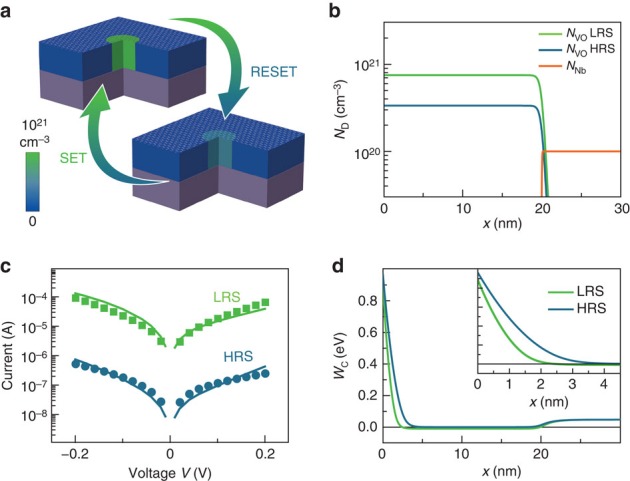
Utilizing PEEM insights for device simulation. (**a**) Schematic of the switching filament in the HRS and in the LRS derived from the spectromicroscopic information. Filament diameter is 500 nm. The colour scale refers to the oxygen-vacancy concentration used for the model as described in **b**. (**b**) Donor distributions as a function of depth *x* used for the simulation of the LRS and the HRS. (**c**) Experimental read-out sweeps (green and blue data points for the LRS and HRS, respectively) of the device in Fig. 3 with simulated *I–V* characteristics based on the model in **a**,**b** (green and blue lines). (**d**) Profiles of the energy of the conduction-band edge *W*_C_(*x*) as a function of depth at zero bias for the LRS and the HRS.

Using the extracted filament dimensions and carrier densities as input into an existing device simulation model^5^, we calculated the *I*–*V* characteristic of the device under investigation in both resistance states, confirming that our findings can yield a comprehensive description of the resistive switching process. For this purpose, we compared simulated *I–V* curves with experimental read-out sweeps at low biases, avoiding larger currents and voltages which might lead to unwanted resistance changes and which would necessitate complex temperature treatments in the simulation. The simulations include drift-diffusion transport of the electronic carriers in the SrTiO_3_ thin film and the Nb:SrTiO_3_ bottom electrode and a Schottky barrier at the top electrode interface (described in detail in ref. 5, Supplementary Table 1 and in Supplementary Methods). As was shown previously for memristive devices with graphene electrodes, we assume that oxygen is removed from the lattice during set and reincorporated during reset^19^. The low-voltage *I*–*V* characteristics of the LRS and HRS are calculated accordingly using static distributions of doubly ionizable donor-type oxygen vacancies in the SrTiO_3_ thin film (Fig. 4a–c). The donor concentrations of both states are derived from the electron densities determined in the XPEEM experiments, that is, the experimentally quantified values serve as an input parameter for the simulations and are not allowed to vary. All other parameters are identical in the simulations of the LRS and the HRS and their values were checked to be physically meaningful. For the reasons of clarity, we chose a uniform donor profile throughout the thin film for this simulation. As the shape of the Schottky barrier is mainly dictated by the donor concentration in the near-interface region, which is determined experimentally in our case, deviations from this uniform donor profile have only minor impact on the *I*–*V* characteristic as shown in Supplementary Fig. 3.

Our results show that spatially confined changes in the donor concentration by a factor of 2–3 at the electrode-oxide interface lead to changes of >2 orders of magnitude in the device resistance (Fig. 4c), induced by the modulation of the effective Schottky barrier height and width (Fig. 4d). Considering the experimental uncertainties and simplicity of the model, the simulation yields a remarkable agreement with the experimental data, validating that the observed redox reaction is indeed responsible for the resistance change. The unprecedented, quantitative agreement between experimental quantification of localized redox reactions and nanoionic device simulation not only clarifies the microscopic origin of the resistance change, but also paves the way for educated design and rational implementation of memristive devices for future memory and logic applications.

We conclude that the resistance change in SrTiO_3_-based devices is indeed caused by a spatially confined redox reaction. This reaction, in turn, leads to a measurable and quantifiable valence change between the HRS and the LRS, confirming the so-called valence change mechanism for resistive switching in transition metal oxides. Through the change of the effective Schottky barrier height and width at the electrode-oxide interface, small donor concentration changes lead to orders of magnitude change in resistance. The direct correlation between the experimental quantitative description of a switching filament by photoemission electron microscopy and nanoionic device simulations provides a significant step towards the design of memristive devices and circuits for applications in future electronics. Beyond this area, photoelectron-transparent graphene electrodes introduced here may find widespread use in the *in operando* analysis of numerous field- or current-driven processes at the nanoscale, such as multiferroics, chemical sensors and electrocatalysts.

## Methods

### Device fabrication

Single-crystalline undoped SrTiO_3_ thin films of 20 nm with 2–3% Sr excess were fabricated via pulsed laser deposition on 0.5 wt% Nb:SrTiO_3_ substrates (CrysTec GmbH, Germany). The single-crystalline SrTiO_3_ target was ablated by a KrF excimer laser (*λ*=248 nm) with a repetition rate of 5 Hz and a spot size of 2 mm^2^ at a target-to-substrate distance of 44 mm. The laser fluence was 1.05 J cm^−2^. All samples were grown in an oxygen atmosphere of 0.1 mbar at a substrate temperature of 800 °C. The film growth was monitored using reflection high-energy electron diffraction.

In a next step, graphene grown by chemical vapour deposition was deposited on the SrTiO_3_ surface as described elsewhere^32^. For the structuring of top electrodes, the graphene layer was patterned through optical lithography and oxygen plasma etching. Before photoresist lift-off, a 30-nm Y:ZrO_2_ insulating layer was deposited via pulsed laser deposition at room temperature. The repetition rate was 5 Hz and the spot size was 1.5 mm^2^ at a target-to-substrate distance of 60 mm in an oxygen atmosphere of 10^−4^ mbar. The laser fluence was 2.1 J cm^−2^. Afterwards, the graphene electrode was partially covered by an additional 80 nm Y:ZrO_2_ insulating layer (optical lithography and pulsed laser deposition). This insulating layer allows for contacting the graphene with Pt/Au leads, which are separated from the continuous bottom electrode. The leads connect to the graphene in one specific position, leaving most of the graphene uncovered, allowing for spectromicroscopic investigation of the SrTiO_3_ layer. The leads are prepared via optical lithography and electron beam evaporation of 10 nm Pt followed by 130 nm Au.

### Electrical characterization

For electrical characterization, the Pt/Au leads were contacted with W whisker probes or through Al wire bonding. The Nb:SrTiO_3_ substrate served as an electrically grounded bottom electrode and was contacted through silver paste. *I*–*V* sweeps were performed with a Keithley 2611A SourceMeter. The different sweeps were performed using the following voltage cycles: 0 V to positive voltages (maximum +5 V) for forming and set, 0 V to negative voltages (maximum −5 V) for reset and +0.2 to −0.2 V for read-out. The device resistance was obtained from the slope of a linear fit of the read-out sweeps between −0.1 and +0.1 V. The step size was 20 mV and the holding time before measurement was 5 ms; the current compliance for the forming step and the set process was 35 mA. During the reset sweeps no current compliance was necessary.

### Spectromicroscopy

The XPEEM experiments have been performed at the beamline UE56/1-SGM at BESSY II (Berlin, Germany) using secondary electrons as detection method. Various series of images were taken at increasing photon energies with a step size of 0.1 eV for the Ti L-edge and 0.2 eV for the O K-edge. The image stacks were analysed and spectra were extracted using the IGOR Pro software. The calculated theoretical resolution of the aberration-corrected instrument in the PEEM mode for 3 eV electrons with Δ*E*=3 eV is 10 nm^33^. The actual resolution can be negatively influenced by imperfect alignment of the microscope and by space charge effects^34^.

For the annealing-reference spectra, a bare SrTiO_3_ thin film was annealed for 15 min at the specified temperatures. After cool down to <100 °C, image stacks were recorded.

For reference spectra from a reduced area, an area exhibiting different amounts of Ti^3+^ was identified and the Ti L-edge spectra were extracted from each point (2 × 2 pixels) of the image stack and numerically described through a linear combination between 60% Ti^3+^ and 0% Ti^3+^ reference spectra, yielding the Ti^3+^ concentration for each point. Points with the same amount of Ti^3+^ were grouped as regions-of-interest and used to extract the spectra displayed in Fig. 3. This procedure yields a direct correlation between Ti^3+^ concentration and the corresponding O K-edge (including the A/B_2_ ratio used for quantification).

All spectra (except for the one shown in Fig. 1c) in the main text and in the Supplementary Information were background corrected by a linear fit of the pre-edge and subsequent normalization by fitting a third-order polynomial to the post-edge using the Athena software^35^. To avoid artifacts from normalization, we always refer to the peak A intensity normalized to the intensity of peak B_2_ at 538 eV. The relative peak ratio trends described here are the same before and after normalization.

### Data availability

The data that support the findings of this study are available from the corresponding author upon request.

## Additional information

**How to cite this article:** Baeumer, C. *et al.* Quantifying redox-induced Schottky barrier variations in memristive devices via *in operando* spectromicroscopy with graphene electrodes. *Nat. Commun.* 7:12398 doi: 10.1038/ncomms12398 (2016).

## Supplementary Material

Supplementary Information Supplementary Figures 1-3, Supplementary Table 1, Supplementary Note 1, Supplementary Methods and Supplementary References
