# Supplementary material for: Quantifying redox-induced Schottky barrier variations in memristive devices via in operando spectromicroscopy with graphene electrodes
Source: Nat Commun. 2016 Aug 19;7:12398. doi: 10.1038/ncomms12398 (PMC4992164; doi:10.1038/ncomms12398)
Supplement: Supplementary Information — Supplementary Figures 1-3, Supplementary Table 1, Supplementary Note 1, Supplementary Methods and Supplementary References [file ncomms12398-s1.pdf]

SUPPLEMENTARY FIGURES

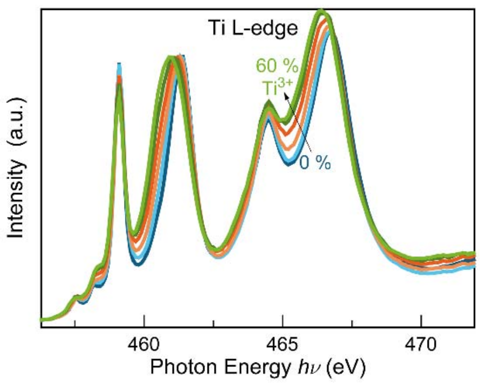

Supplementary Fig. 1: Ti L-edge for increasing Ti^{3+} concentrations. Dark blue: 0 % Ti^{3+}. Light blue: 12 % Ti^{3+}. Orange: 24 % Ti^{3+}. Red: 36 % Ti^{3+}. Dark green: 48 % Ti^{3+}. Light green: 60 % Ti^{3+}.

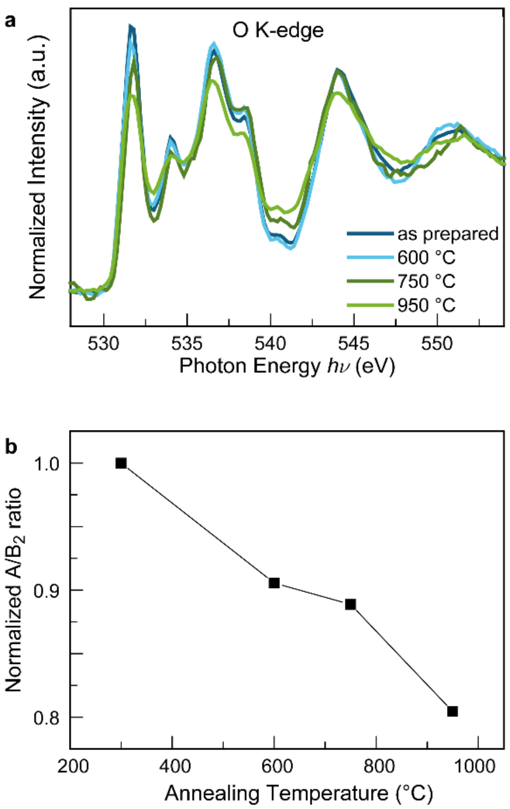

Supplementary Fig. 2: O K-edge in $SrTiO_3$ vacuum annealing series. a) Oxygen K-edge after vacuum annealing at different temperatures. b) Normalized A/B_2 peak ratio for each temperature.

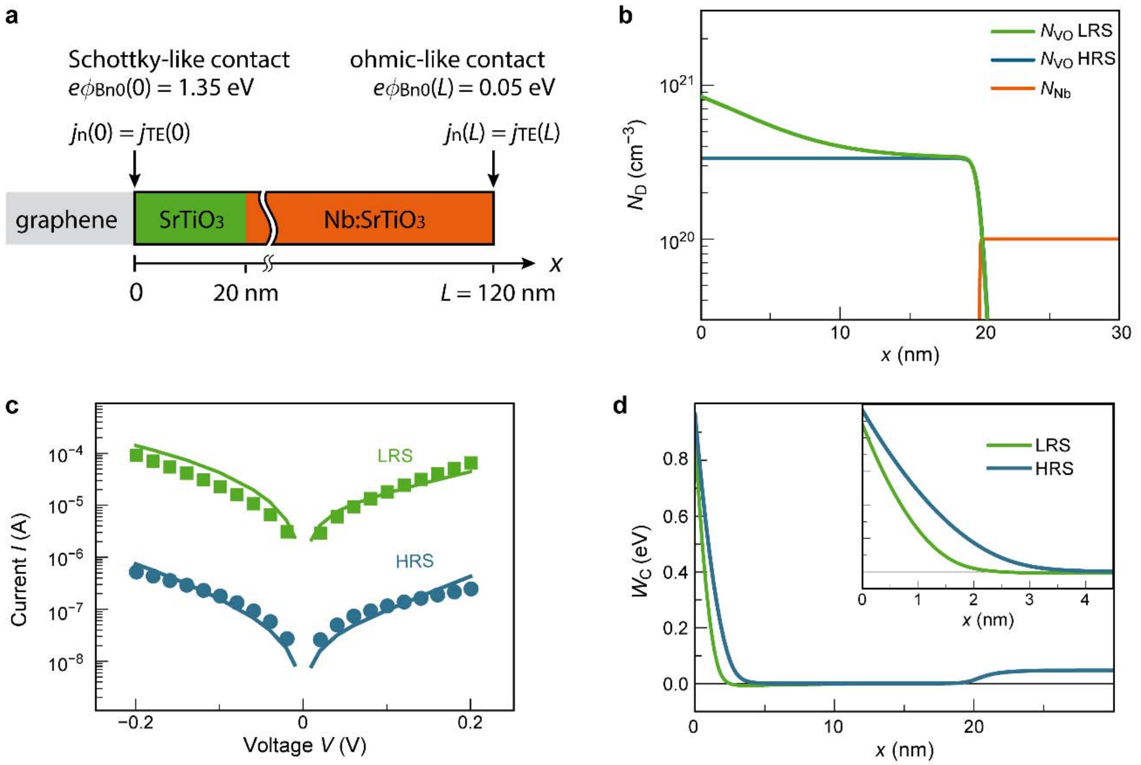

Supplementary Fig. 3: Impact of different donor distributions for the simulation. a) Model geometry. b) Donor distributions for the simulation of the LRS and the HRS. c) Experimental read-out sweeps of the device in Fig. 3 with simulated *I*-*V*-characteristics based on the model in panel b. d) Conduction-band profiles at zero bias-for the LRS and the HRS.

SUPPLEMENTARY TABLES

Supplementary Table 1: Values of the physical parameters used for the simulation of the *I*-*V* curves.

**Symbol**	**Value**	**Symbol**	**Value**
T	300 K	ΔE_g	3.12 eV
N_{VO}^{HRS}	3.35×10^{20} cm^{-3}	$\Delta E_{VO}^{\times\rightarrow1}$	3 meV
N_{VO}^{LRS}	7.5×10^{20} cm^{-3}	$\Delta E_{VO}^{1\rightarrow2}$	30 meV
N_{Nb}	1×10^{20} cm^{-3}	$\Delta E_{Nb}^{\times\rightarrow1}$	50 meV
N_C	2.1×10^{20} cm^{-3}	$\phi_{Bn0}(0)$	1.35 V
N_V	1.3×10^{20} cm^{-3}	$\phi_{Bn0}(L)$	0.05 V
$\varepsilon_{r,opt}$	70	A^*	120 A cm^{-2} K^{-2}
ε_r	5.5	m_t	9.1×10^{-31} kg
μ_n	5.5 cm^2(Vs)$^{-1}$		

SUPPLEMENTARY NOTE 1

The distinctive peaks of the O K-edge stem from the excitation of O 1s core level electrons into unoccupied states of O 2p character[1,2] as given by the selection rules. These states are partly unoccupied due to the hybridization of the O 2p band with metal bands due to partially covalent bonding. The first O K-edge peak (peak A) corresponds to a transition from the O 1s level to a hybrid level between oxygen 2p and Ti t_{2g} states. The intensity of this peak therefore corresponds to the number of unoccupied hybridized Ti t_{2g} states available for the transition from the O 1s level[1]. In the first approximation, we therefore expect a linear decrease of the peak A intensity with the number of electrons in the conduction band[1]. Such a trend was previously observed for a series of different transition metal oxides[1], in aliovalent substitution series of $La_xSr_{1-x}TiO_3$[3] and $Nd_{1-x}TiO_3$[4] as well as in $SrTiO_3$ thin films with oxygen vacancies induced by fabrication[5,6] or *via* electroforming in resistive switching devices[7]. At the same time, the valley between peaks B_2 and C becomes shallower and peak B_2 increases slightly with increasing oxygen vacancy concentration. Compared to the absorption edge around peak A, this change in intensity cannot be easily understood in a simple band-structure model and remains an open question for future investigation. The experimental calibration of the A/B_2 ratio with increasing Ti^{3+} concentration, on the other hand, supplies a valid measure for the charge carrier density.

SUPPLEMENTARY METHODS

The calculation of the *I-V* curves in Fig. 4c of the main text was performed using a one-dimensional numerical model of electron diffusion combined with a Schottky-contact barrier model to account for electron tunneling and thermionic emission across the metal-oxide contacts[8].

The model geometry consists of an oxide layer of length L = 120 nm, representing the 20 nm thick $SrTiO_3$ layer and a 100 nm thick part of the Nb:$SrTiO_3$ bottom electrode as illustrated in Supplementary Fig. 3a . At the contact interface at $x = 0$, a Schottky barrier of nominal barrier height $e\phi_{\mathrm{Bn0}}(0)$ is assumed to be present, while the interface at $x = L$ forms an ohmic contact (low nominal barrier height $e\phi_{\mathrm{Bn0}}(L)$). A donor concentration N_{VO} of doubly ionizable oxygen vacancies is assumed in the $SrTiO_3$ layer, and a donor concentration of homogeneously distributed singly ionizable donors of N_{Nb} is assumed in the Nb:$SrTiO_3$ bottom electrode. All donors are considered immobile throughout the simulations. Further, the temperature is taken to be constant. Neglecting the minority carriers, the Poisson equation for the structure with homogeneous permittivity reads

$$\frac{d^2\psi}{dx^2} = \frac{e}{\varepsilon_0\varepsilon_{\mathrm{r}}}\left(n - N_{\mathrm{VO}}^{+} - 2N_{\mathrm{VO}}^{2+} - N_{\mathrm{Nb}}^{+}\right). \qquad \text{(Supplementary Equation 1)}$$

Here, ψ denotes the electrostatic potential, e the elementary charge, ε_0 the free space permittivity, ε_{r} the relative permittivity of the oxide, n the electron concentration, N_{VO}^{+} (N_{VO}^{2+}) the concentration of the singly (doubly) ionized oxygen vacancies, and N_{Nb}^{+} the concentration of the singly ionized Nb donor. The carrier concentrations n, N_{VO}^{+}, N_{VO}^{2+} , and N_{Nb}^{+} can be calculated as

$$n = N_{\mathrm{C}}\exp\left(-\eta_{\mathrm{Fn}}\right) \qquad \text{(Supplementary Equation 2)}$$

$$N_{\mathrm{VO}}^{+} = \frac{\frac{1}{2}N_{\mathrm{VO}}\exp\left(\eta_{\mathrm{Fn}} - \frac{\Delta E_{\mathrm{VO}}^{\times\to 1}}{k_{\mathrm{B}}T}\right)}{1 + \frac{1}{2}\exp\left(\eta_{\mathrm{Fn}} - \frac{\Delta E_{\mathrm{VO}}^{\times\to 1}}{k_{\mathrm{B}}T}\right)\left(1 + 2\exp\left(\eta_{\mathrm{Fn}} - \frac{\Delta E_{\mathrm{VO}}^{1\to 2}}{k_{\mathrm{B}}T}\right)\right)}, \qquad \text{(Supplementary Equation 3)}$$

$$N_{\mathrm{VO}}^{2+} = \frac{N_{\mathrm{VO}} \exp\left(\eta_{\mathrm{Fn}} - \frac{\Delta E_{\mathrm{VO}}^{\times\to1} + \Delta E_{\mathrm{VO}}^{1\to2}}{k_{\mathrm{B}}T}\right)}{1 + \frac{1}{2}\exp\left(\eta_{\mathrm{Fn}} - \frac{\Delta E_{\mathrm{VO}}^{\times\to1}}{k_{\mathrm{B}}T}\right)\left(1 + 2\exp\left(\eta_{\mathrm{Fn}} - \frac{\Delta E_{\mathrm{VO}}^{1\to2}}{k_{\mathrm{B}}T}\right)\right)}, \quad \text{(Supplementary Equation 4)}$$

$$N_{\mathrm{Nb}}^{+} = \frac{\frac{1}{2} N_{\mathrm{Nb}} \exp\left(\eta_{\mathrm{Fn}} - \frac{\Delta E_{\mathrm{Nb}}^{\times\to1}}{k_{\mathrm{B}}T}\right)}{1 + \frac{1}{2}\exp\left(\eta_{\mathrm{Fn}} - \frac{\Delta E_{\mathrm{Nb}}^{\times\to1}}{k_{\mathrm{B}}T}\right)\left(1 + 2\exp\left(\eta_{\mathrm{Fn}} - \frac{\Delta E_{\mathrm{Nb}}^{1\to2}}{k_{\mathrm{B}}T}\right)\right)}, \quad \text{(Supplementary Equation 5)}$$

with

$$\eta_{\mathrm{Fn}} = \frac{E_{\mathrm{C}} - E_{\mathrm{Fn}}}{k_{\mathrm{B}}T} = \frac{e\left(\varphi_{\mathrm{Fn}} - \psi\right) + \frac{1}{2}\left(\Delta E_{\mathrm{g}} - k_{\mathrm{B}}T \ln\left(\frac{N_{\mathrm{V}}}{N_{\mathrm{C}}}\right)\right)}{k_{\mathrm{B}}T}, \quad \text{(Supplementary Equation 6)}$$

where k_{B} is the Boltzmann constant, T the temperature, $\Delta E_{\mathrm{VO}}^{\times\to1}$ and $\Delta E_{\mathrm{VO}}^{1\to2}$ are the oxygen-vacancy ionization energies, $\Delta E_{\mathrm{Nb}}^{\times\to1}$ is the ionization energy of the Nb donor, E_{C} is the conduction band edge, E_{Fn} the electron quasi-Fermi level in the oxide layer, $\varphi_{\mathrm{Fn}} = -E_{\mathrm{Fn}}/e$ the electron quasi-Fermi potential, ΔE_{g} the band gap, and N_{C} (N_{V}) the effective density of states in the conduction (valence) band.

The electron current density j_n is expressed as

$$j_{\mathrm{n}} = -e\mu_{\mathrm{n}} n \frac{d\psi}{dx} + eD_{\mathrm{n}} \frac{dn}{dx}, \quad \text{(Supplementary Equation 7)}$$

with the electron mobility μ_{n} and the electron diffusion coefficient D_{n}. They are both assumed to be field-independent and to be linked to each other via the Einstein relation

$$\mu_{\mathrm{n}} = \frac{e}{k_{\mathrm{B}}T} D_{\mathrm{n}}. \quad \text{(Supplementary Equation 8)}$$

Along with (6), the steady-state electron continuity equation is applied to solve for n and j_{n}:

$$\frac{dj_{\text{n}}}{dx} = eR_{\text{n}} = \pm\frac{dj_{tunnel}}{dx}. \qquad \text{(Supplementary Equation 9)}$$

Here, the current density due to tunneling through potential barriers j_{tunnel} is converted into a local generation/recombination rate using the procedure outlined in Supplementary Ref. 9. In Supplementary Equation 9, a positive sign in front of the tunneling current-density term corresponds to an annihilation of carriers in front of the tunneling barrier. The tunneling current density through a barrier with potential energy minimum E_{min} and potential energy maximum E_{max} is calculated as

$$j_{\text{tunnel}} = \frac{A^*}{k_{\text{B}}^2}\int_{E_{\text{min}}}^{E_{\text{max}}} T\left(E_x\right) N\left(E_x\right) dE_x, \qquad \text{(Supplementary Equation 10)}$$

where

$$T\left(E_x\right) = \exp\left(-\frac{4\pi}{h}\int_{x_{\text{a}}}^{x_{\text{b}}} \sqrt{2m_{\text{t}}\left(E_C - E_x\right)}\, dx\right) \qquad \text{(Supplementary Equation 11)}$$

is the transmission coefficient as obtained from the Wentzel–Kramers–Brillouin approximation and

$$N\left(E_x\right) = k_{\text{B}}T \ln\left(\frac{1+\exp\left(-\dfrac{E_x - E_{\text{Fn}}\left(x_{\text{a}}\right)}{k_{\text{B}}T}\right)}{1+\exp\left(-\dfrac{E_x - E_{\text{Fn}}\left(x_{\text{b}}\right)}{k_{\text{B}}T}\right)}\right) \qquad \text{(Supplementary Equation 12)}$$

is the supply function. In the above equations, A^* is the effective Richardson constant, h is the Planck constant, x_{a} and x_{b} are the classical turning points, and m_{t} is the electron tunneling mass.

The current density of thermionic emission j_{TE} at the geometry boundaries $x_{\text{i}} = \{0, L\}$ is formulated analogously to the tunneling current density in Supplementary Equation 10. With a transmission coefficient of 1 for charge carrier emission over the barrier and the upper integration limit of infinity, one obtains the boundary conditions for the solution of the electron continuity equation:

$$j_{\mathrm{TE}}(x_i)=\frac{A^*T}{k_{\mathrm{B}}}\int_{E_{\mathrm{C}}(x_i)}^{\infty}\ln\left(\frac{1+\exp\left(-\frac{E_x-E_{\mathrm{F,a}}(x_i)}{k_{\mathrm{B}}T}\right)}{1+\exp\left(-\frac{E_x-E_{\mathrm{F,b}}(x_i)}{k_{\mathrm{B}}T}\right)}\right)dE_x. \qquad \text{(Supplementary Equation 13)}$$

Here, it is $E_{\mathrm{F,a}}(x_{\mathrm{i}}) = \{E_{\mathrm{F,M1}}, E_{\mathrm{Fn}}(L)\}$ and $E_{\mathrm{F,b}}(x_{\mathrm{i}}) = \{E_{\mathrm{Fn}}(0), E_{\mathrm{F,M2}}\}$ with $E_{\mathrm{F,M1}}$ ($E_{\mathrm{F,M2}}$) being the metal Fermi level of the top (bottom) contact.

The boundary conditions for the potential are expressed in terms of applied potential $\psi_{\mathrm{a}}(x_{\mathrm{i}})$ at the electrodes and the potential barrier heights $\phi_{\mathrm{Bn0}}(x_{\mathrm{i}})$ at the contacts. The potential at the contact interface is given as

$$\psi(x_i)=\psi_{\mathrm{i}}^0+\frac{k_{\mathrm{B}}T}{e}\eta_{\mathrm{Fn}}^0-\phi_{\mathrm{Bn}}(x_i)+\psi_{\mathrm{a}}(x_i), \qquad \text{(Supplementary Equation 14)}$$

with $\psi_{\mathrm{i}}^0(x_{\mathrm{i}})$ being the intrinsic potential and η_{Fn}^0 the energy difference between the bottom of the conduction band and the Fermi level in the undisturbed oxide, normed by $k_{\mathrm{B}}T$. The term $\phi_{\mathrm{Bn}}(x_{\mathrm{i}})$ denotes the effective potential barrier height at the contact that results from including image-force-induced barrier lowering by an amount $\Delta\phi_{\mathrm{Bn}}(x_{\mathrm{i}})$ (Schottky effect). It is described as

$$\phi_{\mathrm{Bn}}(x_i)=\phi_{\mathrm{Bn0}}(x_i)-\Delta\phi_{\mathrm{Bn}}(x_i)\approx\phi_{\mathrm{Bn0}}(x_i)-\sqrt{\frac{e\left|\frac{d\psi(x_i)}{dx}\right|}{4\pi\varepsilon_0\varepsilon_{\mathrm{opt}}}} \qquad \text{(Supplementary Equation 15)}$$

with the optical relative dielectric constant $\varepsilon_{\mathrm{r,opt}}$. With the right hand side of Supplementary Equation 15 substituted for $\phi_{\mathrm{Bn}}(x_{\mathrm{i}})$, Supplementary Equation 14 is transformed into

$$\frac{d\psi(x_i)}{dx}=-\frac{4\pi\varepsilon_0\varepsilon_{\mathrm{opt}}}{e}\left[\psi(x_i)-\left(\psi_{\mathrm{i}}^0+\frac{k_{\mathrm{B}}T}{e}\eta_{\mathrm{Fn}}^0-\phi_{\mathrm{Bn0}}(x_i)+\psi_{\mathrm{a}}(x_i)\right)\right]^2, \qquad \text{(Supplementary Equation 16)}$$

yielding nonlinear Robin boundary conditions for the potential at the contact interfaces. The total current density in the structure can be computed as the drift-diffusion current density according to Supplementary Equation 7 at a location x of minimal conduction-band energy.

To calculate the current-voltage curves, the simultaneous solution of the Poisson equation (Supplementary Equation 1) and the continuity equation for electrons (Supplementary Equation 9) is computed for different applied voltages $V_a = \psi_a(0) - \psi_a(L)$ as follows: With η_{Fn} expressed in terms of ψ and φ_{Fn} according to Supplementary Equation 6, Supplementary Equations 2–5 are substituted for the charge carrier concentrations in Supplementary Equation 1. For a fixed value of φ_{Fn}, the resulting nonlinear differential equation is solved for the potential using a Newton-Raphson scheme with the boundary conditions Supplementary Equation 16 being linearized and updated in each iteration step. The new values for ψ and the corresponding values for E_C are used in Supplementary Equation 7, 10, 11, and 13 during the solution of the continuity equation (Supplementary Equation 9). Due to the dependence of the supply function on the quasi-Fermi level or corresponding carrier concentration, the resulting differential equation is as well linearized and iteratively solved in a Newton-Raphson loop to obtain n and φ_{Fn}. This procedure of consecutively solving Supplementary Equation 1 and Supplementary Equation 9 is repeated until self-consistency is reached.

As described in the main text, we calculated *I-V* characteristics for the LRS and the HRS assuming different oxygen-vacancy distributions in both states, denoted by N_{VO}^{LRS} and N_{VO}^{HRS}. The concentration values and other parameters used in the simulations are given in Supplementary Table 1.

As there is experimental evidence of a concentration gradient near the Schottky-contact interface in the LRS [David Cooper, CEA LETI, Grenoble, France; unpublished results], we also simulated the *I-V* characteristic for the case of a non-uniform oxygen-vacancy distribution in the LRS shown in Supplementary Fig. 1b. Here, the average concentration over the first 3 nm is taken to be 7.5×10^{20} cm^{-3}, which is the value obtained from the PEEM experiments and the same value that was used for the calculations with the homogeneous profile shown in Fig. 4b. Further, the concentration is assumed to decrease to the value of N_{VO}^{HRS} in direction of the bottom electrode interface. For the HRS, the same donor profile as presented in the main document (Fig. 4b) was used. As evident from Supplementary Fig. 3c, the simulated *I-V* characteristic of the inhomogeneous LRS provides a good match to the experimental data,

similar to the one obtained for the homogeneous oxygen-vacancy profile. These findings indicate that the barrier width and height (Supplementary Fig. 3d) is primarily determined by the concentration in vicinity of the Schottky-barrier interface. For a fixed average concentration in this region, the presence of a concentration gradient within the $SrTiO_3$ layer only has a small impact on the *I*-*V* characteristic. We therefore conclude that the small change of concentration (by a factor 2-3) in the near-interface region only is able to induce a current modulation by more than two orders of magnitude, resulting in distinct resistances of the LRS and the HRS.

SUPPLEMENTARY REFERENCES

1. de Groot, F. M. F. *et al.* Oxygen 1 s X-ray-absorption edges of transition-metal oxides. *Phys. Rev. B* **40,** 5715–5723 (1989).

2. de Groot, F. *et al.* Oxygen 1s X-ray absorption of tetravalent titanium oxides: A comparison with single-particle calculations. *Phys. Rev. B* **48,** 2074–2080 (1993).

3. Fujimori, A. *et al.* Doping-induced changes in the electronic structure of $La_xSr_{1-x}TiO_3$: Limitation of the one-electron rigid-band model and the Hubbard model. *Phys. Rev. B* **46,** 9841–9844 (1992).

4. Sefat, A. S., Amow, G., Wu, M.-Y., Botton, G. A. & Greedan, J. E. High-resolution EELS study of the vacancy-doped metal/insulator system, Nd1−xTiO3, to 0.33. *J. Solid State Chem.* **178,** 1008–1016 (2005).

5. Filatova, E. O. *et al.* X-ray spectroscopic study of $SrTiO_x$ films with different interlayers. *J. Appl. Phys.* **113,** 224301 (2013).

6. Shah, A. B. *et al.* Probing interfacial electronic structures in atomic layer $LaMnO_3$ and $SrTiO_3$ superlattices. *Adv. Mater.* **22,** 1156–1160 (2010).

7. Andreasson, B. P. Oxygen Vacancies in $SrTiO_3$: An X-ray Absorption Study. PhD Thesis, ETH ZÜRICH (2009).

8. Marchewka, A., Waser, R. & Menzel, S. Physical simulation of dynamic resistive switching in metal oxides using a Schottky contact barrier model. in *2015 Int. Conf. Simul. Semicond. Process. Devices* 297–300 (IEEE, 2015). doi:10.1109/SISPAD.2015.7292318

9. MeiKei Ieong, Solomon, P. M., Laux, S. E., Wong, H.-S. P. & Chidambarrao, D. Comparison of raised and Schottky source/drain MOSFETs using a novel tunneling contact model. in *Int. Electron Devices Meet. 1998. Tech. Dig. (Cat. No.98CH36217)* 733–736 (IEEE). doi:10.1109/IEDM.1998.746461
